# Evolutionary analysis of the most polymorphic gene family in *falciparum* malaria

**DOI:** 10.12688/wellcomeopenres.15590.1

**Published:** 2019-12-03

**Authors:** Thomas D. Otto, Sammy A. Assefa, Ulrike Böhme, Mandy J. Sanders, Dominic Kwiatkowski, Pf3k consortium, Matt Berriman, Chris Newbold

**Affiliations:** 1 Parasite Genetics, Wellcome Trust Sanger Institute, Hinxton, UK; 2 Institute of Infection, Immunity & Inflammation, MVLS, University of Glasgow, Glasgow, UK; 3 The Wellcome Trust Centre for Human Genetics, University of Oxford, Oxford, UK; 4 Weatherall Institute of Molecular Medicine, University of Oxford, John Radcliffe Hospital, Oxford, UK

**Keywords:** Plasmodium, var, evolution

## Abstract

The *var *gene family of the human malaria parasite *Plasmodium falciparum* encode proteins that are crucial determinants of both pathogenesis and immune evasion and are highly polymorphic. Here we have assembled nearly complete *var *gene repertoires from 2398 field isolates and analysed a normalised set of 714 from across 12 countries. This therefore represents the first large scale attempt to catalogue the worldwide distribution of *var* gene sequences

We confirm the extreme polymorphism of this gene family but also demonstrate an unexpected level of sequence sharing both within and between continents. We show that this is likely due to both the remnants of selective sweeps as well as a worrying degree of recent gene flow across continents with implications for the spread of drug resistance. We also address the evolution of the *var* repertoire with respect to the ancestral genes within the *Laverania* and show that diversity generated by recombination is concentrated in a number of hotspots. An analysis of the subdomain structure indicates that some existing definitions may need to be revised

From the analysis of this data, we can now understand the way in which the family has evolved and how the diversity is continuously being generated. Finally, we demonstrate that because the genes are distributed across the genome, sequence sharing between genotypes acts as a useful population genetic marker.

## Introduction

*Plasmodium falciparum* is the most virulent of the human malaria parasites, at least in part because it can concentrate in the small vasculature of organs via its ability to adhere to endothelial cells leading to organ dysfunction. It has also developed the ability to maintain chronic infections in humans to maximise the probability of mosquito transmission. A single multi-gene family of ~60 *var* genes in this parasite is a major determinant of both of these important phenotypes. The *var* genes encode a family of proteins called *Plasmodium falciparum* Erythrocyte Membrane Protein 1 (PfEMP1) that are expressed on the surface of infected red cells in a mutually exclusive fashion ^1^. On the red cell surface, PfEMP1 proteins mediate both adherence to endothelium and induce host protective antibodies. The latter are evaded through transcriptional switches among the *var* gene family such that new sequences are periodically expressed. Through their role in immune evasion they have evolved to be extremely polymorphic. The genes are located mostly in subtelomeric regions but are also located in a few chromosome-internal clusters. Analysis of fully sequenced genes has shown that *var* genes can be subdivided into three major classes (UPS A, B and C), based on their upstream sequences and chromosomal position, and that they are composed of long 5’ exons that encode multiple combinations of two types of extracellular domains, the Duffy Binding Like (DBL) and Cysteine Rich Interdomain Regions (CIDR) and a more conserved 3’ exon encoding the intracellular region ^2, 3^. The DBL and CIDR regions have been divided into subclasses based on sequence similarity ^2, 3^. Most of the data from field isolates on *var* gene sequences has to date been collected by PCR of a small region of DBLα, eg: ^4– 9^. Analysis of many thousands of such amplicons first produced a classification system for members of this domain into six classes based on cysteine content, amplicon length and the presence of certain sequence motifs ^10^. Surprisingly, considering the fact that these amplicons only represented a tiny fraction of the total gene sequence, associations have been found with the expression of particular DBLα types and parasite phenotype ^11, 12^. A more extensive analysis of full-length genes from the sequenced genomes of seven laboratory-adapted isolates revealed that all domain subclasses can be further divided into a number of subdomain types and that in some cases these can be grouped together into “domain cassettes” ^13^. Some of these domain cassettes (DC) are associated with binding to specific receptors on endothelial cells; for instance, *var* genes containing DC8 and 13 are expressed in parasites causing severe malaria and bind to endothelial protein C receptor (EPCR) ^14^. More recently, a combination of Illumina and long read Pacific Biosciences sequencing has been used to assemble the genomes, including the *var* genes from 12 Malian isolates ^15^ and 15 field isolates ^16^. This has confirmed that the number and domain organisation of *var* genes in these samples is similar to that in the 3D7 reference. RNAseq of parasites isolated from severe and non-severe patients in Indonesia has also been used to assemble expressed *var* gene sequences ^17^.

The *var* gene family is restricted to the *Laverania* sub-genus of malaria parasites that infect great apes and from which *P. falciparum* evolved. However, the fact that the number, genomic location and domain architecture of these genes is very similar in *P. reichenowi* (a parasite of chimpanzees that diverged from the *P. falciparum* lineage around 200,000 years ago) suggests that the evolutionary fitness of the diversity and organisation of this family has been long established ^18^.

The central role of these genes in crucial areas of malaria biology has meant that they have been the subject of extensive research interest, but to date the paucity of full-length sequence information has precluded an in-depth global study. Recently, large scale sequencing has produced short-read data for thousands of *P. falciparum* isolates but to date the analysis of these data has been restricted to coding sequences within the central core genome and has not addressed the sub-telomeres or central *var* gene clusters ^19^. Here we have assembled almost complete *var* gene repertoires from MalariaGEN *P. falciparum* Community Project and *Pf3k* datasets and present data from 12 countries for which ~60 isolates were available from each. Using this normalised dataset, we performed a detailed analysis of *var* gene sequence-sharing across the world. Sharing across the dataset is much greater than had been expected and there were clear hotspots of recombination. Subdomains of DBL and CIDR sequences could be identified based on their sequence conservation, but we show that some current definitions are not robust. Many of the sequences that comprise extant *var* genes can also be traced back to their ancestors in great apes, and because *var* genes are distributed throughout the genome, we demonstrate that these genes are rich and informative population genetic markers for the genome as a whole.

## Results

Using whole genome sequencing data from approximately 2,400 clinical isolates of *Plasmodium falciparum* produced by the Pf3K project ( *Extended data*: Table S1), we developed a pipeline (Methods) that enabled us to assemble contigs representing putative individual *var* exons. Throughout this study, a *var* assembly refers to its large exon I sequence, rather than the whole gene that also includes a highly conserved and much smaller exon II sequence. The number of LARSFADIG motifs (the only motif shared by all DBLα domains that are present in exon I of all regular *var* genes) is a proxy for the number of *var* genes/genome. To create a normalised dataset, we included 714 samples containing between 45 and 90 LARSFADIG motifs to minimise the number of multiple infections present ( Table 1). All genomes in our normalised set contained variable numbers of *var* pseudogenes that inevitably assembled as short fragments and did not contribute to the accurate number of intact genes assembled. By focussing on *var* genes > 3.5 kb, we observed approximately the expected number of *var* genes/genome with a tendency for a higher number from African countries where higher transmission increases the probability of infection with multiple genotypes. This was confirmed in 20 by the presence of multiple genotypes of polymorphic single copy genes. To further validate the data, we used our previously assembled genome sequences of 12 isolates that had been adapted to culture to provide enough DNA for long-read PacBio sequencing ^16^. The PacBio assemblies were corrected with short-read sequencing produced using Illumina HiSeq 2500 and the *var* genes were subsequently manually curated. Comparison of our assembled Illumina and PacBio data did show one systematic error: in 1.5% of *var* exon I sequences, frame-shifts occurred within homo-polymer tracts that could not be corrected by iterative alignment and error-correction using iCORN ^21^. As a consequence, we estimated that our assemblies are 98.5% accurate, vastly exceeding the level required for our downstream analysis of high frequency events.

**Table 1.** Summary of normalised dataset. *var1CSA* and *var2CSA* sequences and exon2 are excluded.

Country of origin	Analysed isolates *	Average number of LARSFAGID motifs per genome	Average number of sequences > 3kb per genome	Unique shared *var* genes per genome **
The Gambia	60	63.6	62.1	20.6
Kenya	55	50.5	48	6.3
Thailand	60	55.1	54.5	33.2
Ghana	60	63.3	62.6	6.6
Cambodia	60	56.1	53.6	29.6
Mali	60	70.3	70	8.6
Senegal	60	51.2	46.8	8.6
Malawi	60	65.5	61.5	6.1
Guinea	60	59.4	62.6	8.1
Vietnam	59	57.2	50.3	21.0
Laos	60	58.9	58.5	27.1
Congo	60	63.2	60.3	9.2

* Genomes excluded due to contamination or not enough assembled sequences ** unique count of hit against normalised set, hit > 99% identity, > 3.5kb, 80% overlap

### Polymorphism

**Figure 1.**
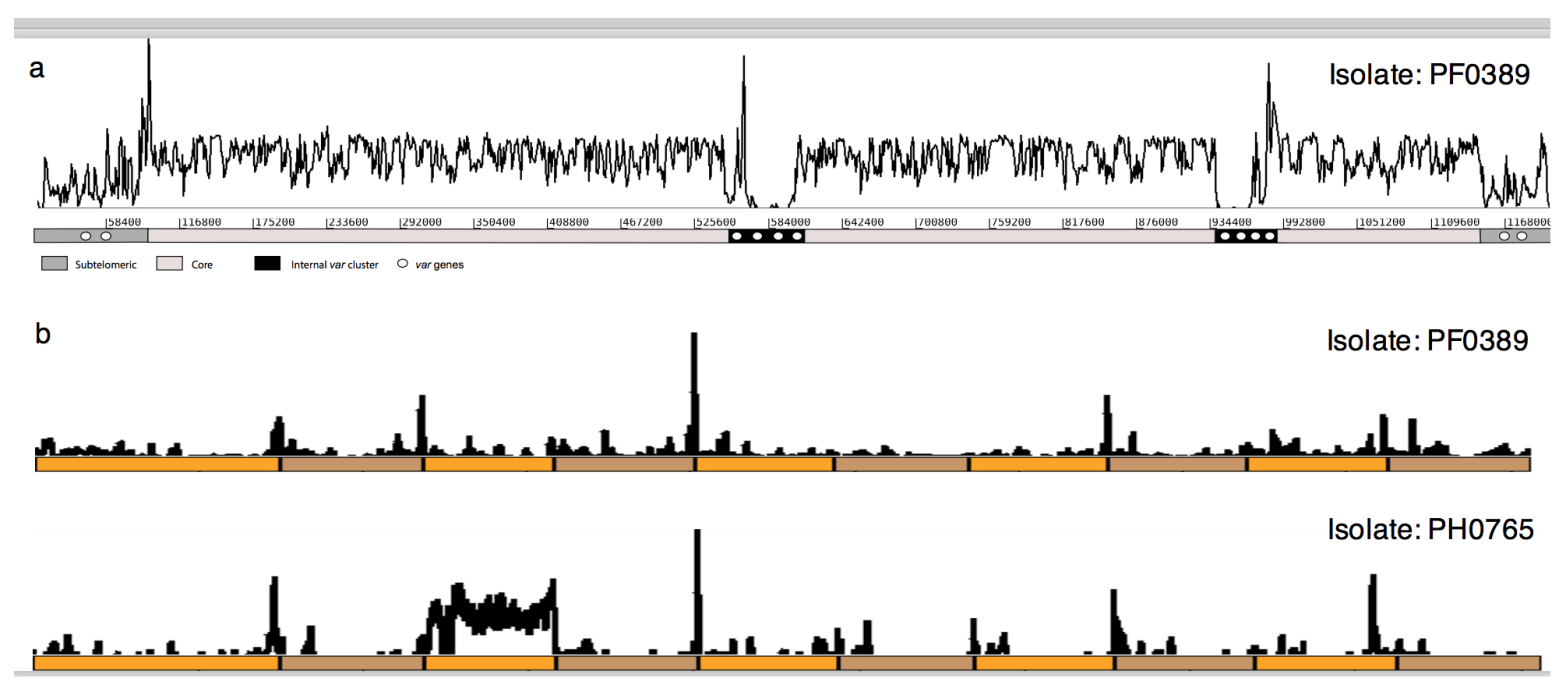
Mapped-coverage of sequencing reads from clinical isolates. ( **a**) Sequencing reads from a clinical isolate (PA0274) map at high coverage across much of chromosome 4 of the *P. falciparum* 3D7 reference genome. The distal regions of the subtelomeres and discrete interstitial regions contain *var* genes and are clear exceptions with little or no coverage, indicating high sequence polymorphism. ( **b**) Sequencing reads from the PF0389 (upper) and PH0765 (lower) clinical isolates mapped against a reference comprising 10 concatenated *var* genes from *P. falciparum* 3D7 (alternating orange/brown). Few regions of the *var* genes are similar enough between isolates to enable reads to map and just one *var* gene is completely covered in one clinical isolate.

The degree of polymorphism in the assembled *var* genes can be shown by mapping the short sequence reads back to the 3D7 reference sequence ( Figure 1a). The areas of very low or highly variable sequence-coverage coincide with the positions of *var* genes. Thus, when the reads from one of our sequenced isolates were mapped to a random set of ten *var* sequences from the genome-reference clone 3D7, similarly patchy coverage was seen ( Figure 1b, top panel). Given the known highly polymorphic nature of *var* gene sequences, we were surprised to observe that in some cases ( Figure 1b, bottom panel) reads from a field isolate mapped almost completely to a single 3D7 gene; a gene from the reference clone was therefore almost completely duplicated in an unrelated field isolate.

### Conserved *var* genes

**Figure 2.**
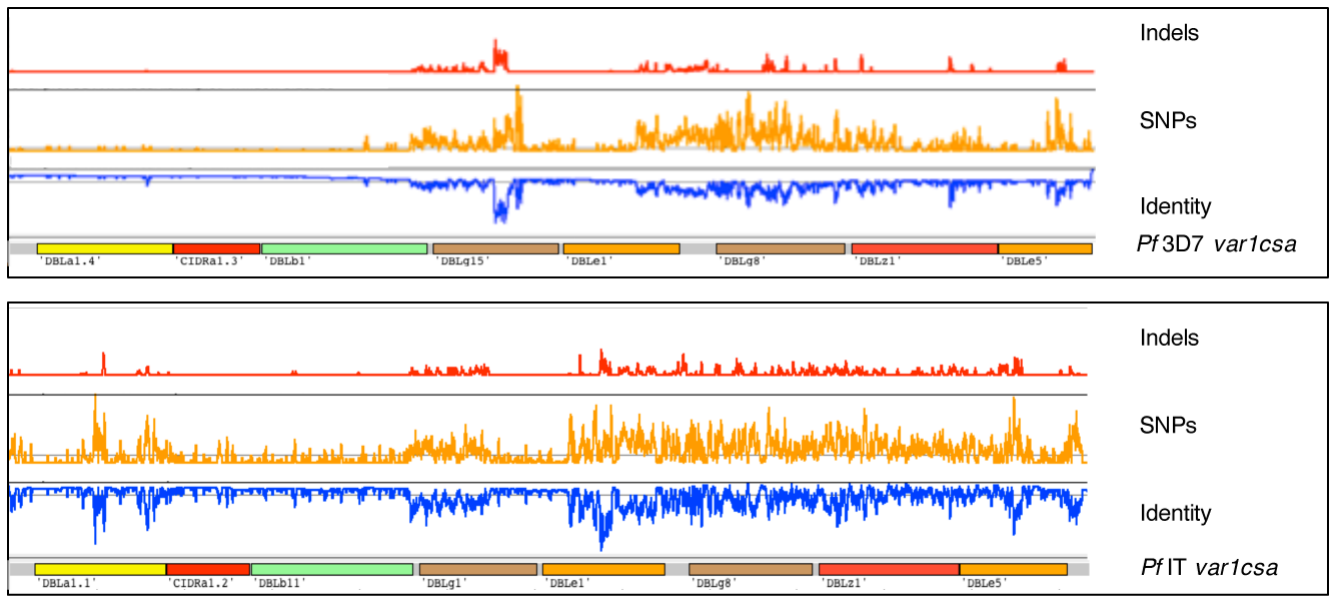
Sequence diversity of *var1CSA* genes from clinical isolates. *var1CSA* genes were split into two major types corresponding to *P. falciparum* 3D7 (top) and *P. falciparum* IT (bottom) *var1CSA* reference genes, based on a phylogenetic analysis (Addition File 2: Figure S1). Using BWA-MEM and SAMtools Pileup, sequence identity and polymorphism were detected and then plotted across each reference. The regions of each gene encoding specific protein domains are indicated. Genes of the 3D7 type have high sequence identity across their entire length but those of the IT type show greater polymorphism, particularly in their 3’ half.

Three types of *var* gene are known to be unusual in having greater sequence similarity to their orthologues than the repertoire as a whole: *var1CSA* is expressed late in the cell cycle and does not appear to reach the red cell surface ^22^, *var2CSA* has an atypical domain architecture, is most expressed in infected women in their first pregnancy and mediates binding to chondroitin sulphate in the placenta resulting in low birth weight and premature delivery ^23^, *var3* is a short gene of unknown function, of which there are three highly conserved copies in the 3D7 reference and which have been shown to be expressed on the infected red cell surface ^24^. Before carrying out any further analysis on our global *var* set, we therefore extracted these unusual *var* sequences. We found that *var1CSA* is universally present as two distinct allelic forms that resemble the sequences of 3D7 (38%) or IT (62%) and show no obvious evidence of recombination. The 5’ region of the 3D7 allele showed almost complete sequence conservation (only 0.5% divergence) compared with the same 3.2 kb region of the IT allele (3.6% divergence; Figure 2). A maximum likelihood tree drawn from the full-length amino acid sequences of *var1CSA* from all 714 isolates reflected the overall bi-allelic structure but showed more complex branching ( *Extended data*: Figure S1). There appears to be no significant change in the ratio of allelic types by region and indeed the same bi-allelic pattern can be traced back more broadly to the *Laverania* subgenus from which *P. falciparum* emerged ^25^.

Global sequence diversity and analysis in *var2CSA* has been recently reported for 1,249 isolates from the MalariaGEN data ^26^ and so will not be described in detail here. However, in addition to the conclusions of these authors, we found very elongated *var2CSA* sequences of up to 15kb in some isolates (eight >12kb), and some similar extended sequences within the PacBio assemblies. The additional sequences are composed primarily of a single domain type, DBL epsilon ( *Extended data*: Figure S2). Within this latter subset we find four sequences from South East Asia that share >99% identity with a 6kb region of the orthologue from *P. praefalciparum*, a parasite of gorillas that is the closest relative of *P. falciparum*.

The three *var3* genes in 3D7 show between 96–98% identity. In our normalised dataset we find 680 copies from 714 isolates with ~98% identity suggesting both a high degree of sequence conservation and degree of copy number variation. Indeed, within the PacBio dataset the number of copies varies from zero to six.

### Shared *var* sequences

**Figure 3.**
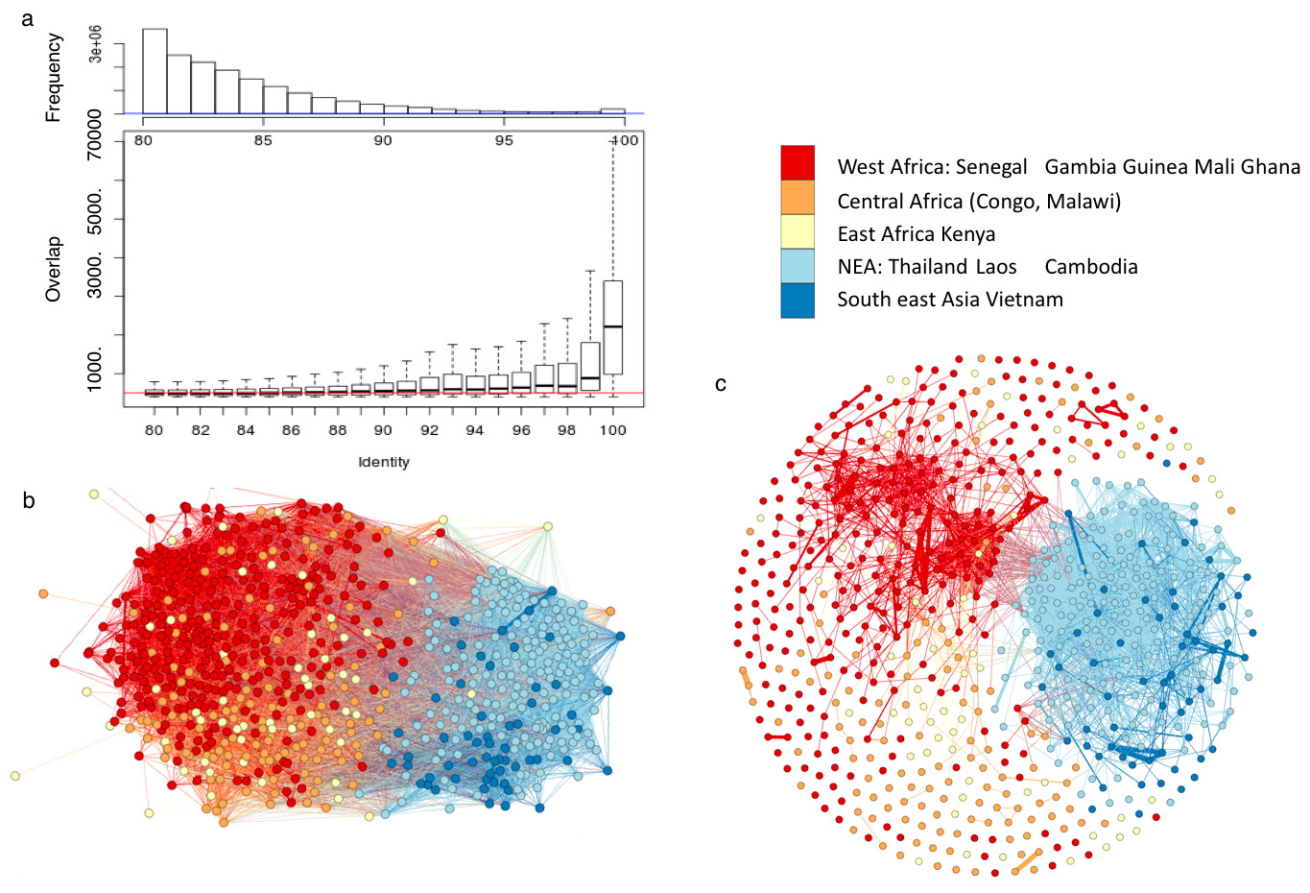
Extensive sequence sharing between *var* genes. ( **a**) Boxplot of nucleotide-alignment lengths versus sequence identity between *var* genes. ( **b**) Network of *var* sharing between normalized dataset of 714 isolates. Each node represents an isolate, coloured by region. Edges represent isolates sharing at least one *var* gene (> 99% identity, 3.5 kb overlap and > 80% sequence overlap). ( **c**) Alternative network of *var* sharing but with nodes (isolates) connected with three shared *var* genes.

Having excluded genes known to have similar sequences, the most striking feature of the data, bearing in mind the highly polymorphic and recombinogenic nature of these genes, was the presence of long stretches of nucleotide sequence that are almost perfectly shared between *var* sequences of different isolates. For instance, using stringent alignment criteria (>3.5 kb alignments with >99% identity and >80% overlap, used throughout this study unless otherwise stated) we identified 59,202 pairwise matches within the dataset representing 11,054 genes. We therefore reasoned that sharing of these polymorphic gene sequences at almost base perfect levels must have arisen from recent recombination events. Examining the length of the matches against their sequence similarity revealed a sharp decay in the length of matches when sequence identity dropped below 100% ( Figure 3a). The longer matches must therefore represent recent events that have not had time to undergo recombination or SNP accumulation.

As a more visual representation of the way in which stretches of *var* gene sequence are shared between isolates, we constructed networks from a subset of the data where nodes (isolates) were connected if they shared a single *var* gene sequence using the same alignment criteria ( Figure 3b). The same sequences could clearly be seen to be present across different continents showing an unexpected level of gene flow across the world. This analysis however hides a degree of granularity in the data (shown in Figure 3c) where nodes are only connected if they share at least three *var* gene sequences. At this more stringent level, the sharing of sequences between isolates is now dominated by South East Asian isolates and by two small groups of mainly African isolates. South East Asia is discussed in more detail later.

Following a more global approach to quantitate the level of sequence sharing, we clustered all of the sequences based on their top similarity hit using OrthoMCL and generated 3,351 clusters containing the 11,054 stringently aligned genes. After classifying the dataset into large geographic areas (West Africa, East and Central Africa, and Asia), we found 26 of the clusters containing 419 genes were present across all areas and 92 clusters containing 508 genes were shared between at least one African and one Asian area. Not unexpectedly, when we decreased the minimum length of the genes within a cluster to >2 kb, there were far more (241) clusters containing 22,466 genes of which 4,002 were present in all areas of Africa and Asia ( *Extended data*: Table S2), reinforcing the conclusion that there is significant intercontinental gene flow.

To gain some insight into the distribution and chromosomal position of these shared *var* genes, all of the complete *var* gene sequences from our PacBio assemblies were BLAST-searched against our database and genes of >3.5 kb with ≥ 99% identity were identified. A small proportion of genes from the PacBio-sequenced isolates (3.2%) had a comparatively large number of hits (up to 55) and 81% of these highly shared genes, with >10 hits, were concentrated primarily in internal clusters on chromosomes 4 and 7 and to a lesser extent chromosomes 8 and 12 as well as a subtelomeric gene on chromosome 6 ( Table 2). In the former case, they appear to be a consequence of a selective sweep by chloroquine resistance on chromosome 7 ^27^. For reasons that are not entirely clear, the signature of the selective sweep has not been eliminated by recombination in a subset of isolates (22) resulting in a 350 kb region around the selected locus including four *var* genes remaining shared ^25^. Similar arguments may apply to selective sweeps by anti-folate drugs on chromosomes 4 and 8. The reasons for an apparent additional selective sweep on chromosome 6 are unclear, but the sweep has been noted previously from SNP data ^28^.

**Table 2.** The chromosomal position of *var* genes. Only *var* genes from the global set, with >10 matches (> 3.5 kb, with > 99% identity and 80% overlap) to a *var* gene from twelve PacBio-based *P. falciparum* assemblies were counted.

Chromosome	>10 hits	%
Chr 7	19	32.8
Chr 4	16	27.6
Chr 6	11	19.0
Chr 12	5	8.6
Chr 8	5	8.6
Other Chromosome	7	12.1
No data	2	3.4
Total	65	100.0

**Figure 4.**
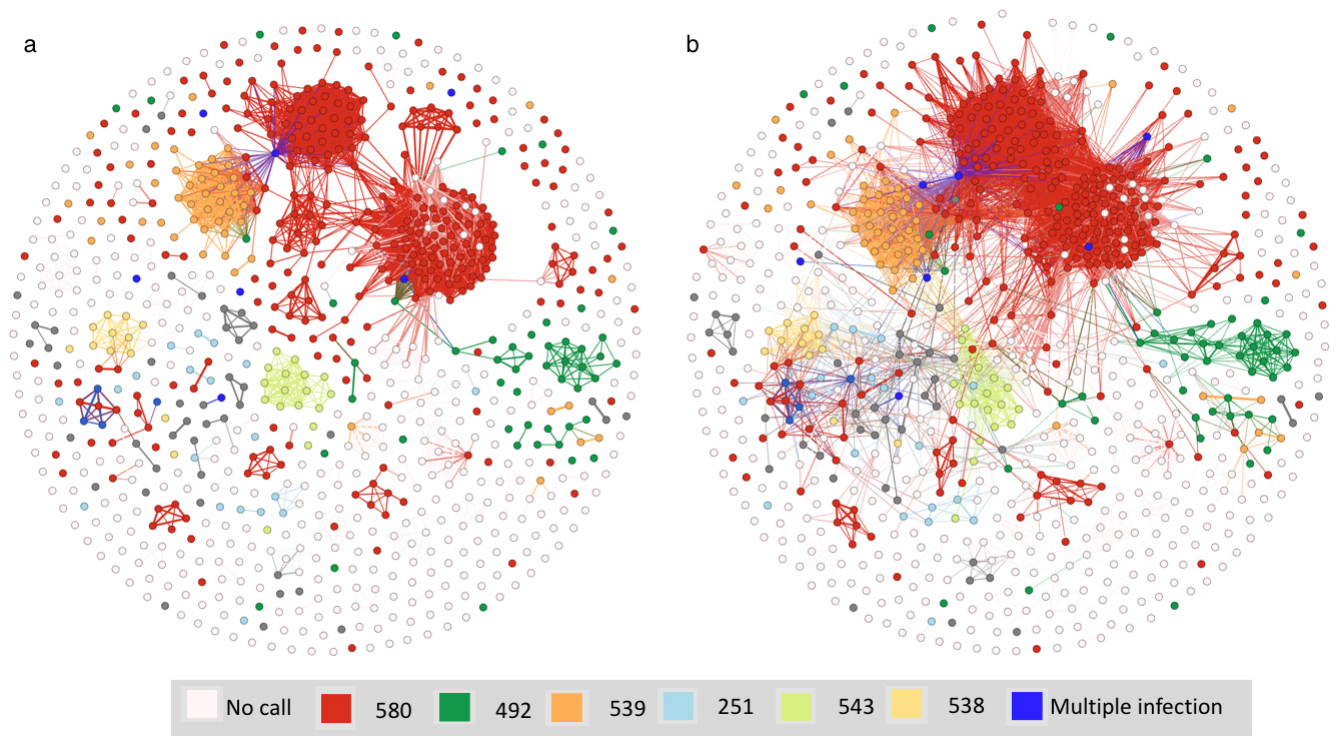
*var* genes record pattern of artemisinin resistance in SE Asia. Network of *var* sharing with each node representing an isolate, coloured based on polymorphisms in the *kelch13* gene. Edges represent either ( **a**) ≥ 15 shared *var* genes (99% identity, ≥ 3.5kb and 80% overlap), or ( **b**) ≥ 7 shared *var* genes. Dark blue isolates are where more than one SNP occurs in *kelch13.* Additional samples were used for this figure (see methods).

Sequence sharing is most striking amongst the SE Asian isolates ( Table 1). In Cambodia, a distinct population substructure has arisen in which resistance to the drug Artemisinin (through point mutations in the Kelch gene) was associated with the expansion of non-interbreeding subpopulations from a recent bottleneck. To determine whether the level of *var*-sequence sharing is due to this recent bottleneck, we built a network from the SE Asian assemblies ( Figure 4) clustered by the Kelch mutation that the isolates contained. Moreover, by relaxing the network inclusion criteria from >15 *var* genes to >7, we were able to see likely mixed infections where different Kelch mutations may have come together. The bottleneck that produced these different populations probably occurred around 20 years ago with the introduction of Artemisinin, providing a useful baseline for tracking *var* gene evolution over time and for calibrating the relationship demonstrated in Figure 3a ( *Extended data*: Figure S3).

### Gene duplication

*Var* gene duplication within a single genotype has been observed in cultured isolates. We performed the analysis on the 12 full PacBio assembled genomes ( *Extended data*: Table S3) and found between 4–21 duplications of at least 2kb. However, within our assembly process, such duplications would be assembled as single genes. We therefore examined intra-genome duplication by an isolate specific analysis of read depth. We chose samples containing a single genotype (defined as containing between 45 and 90 LARSFADIG sequences). We found a mean of 1.35 *var* genes/genome containing a partial duplication (range 0–10) and an average of 0.6 *var* genes/genome that were completely duplicated (range 0–8). These frequencies varied between Asian and African samples where we found slightly more duplication in the former (1.54 vs 1.19 per genome for partial duplication and 0.6 vs 0.54 per genome for complete duplicates). Thus, intra-genome duplication events are relatively common and more frequent in areas of more limited genetic diversity. Whether this reflects an increased rate of inbreeding or is related to a higher *var* gene recombination rate in Asian parasites remains to be determined. Partial duplication indicates a meiotic or mitotic recombination event.

### Recombination

**Figure 5.**
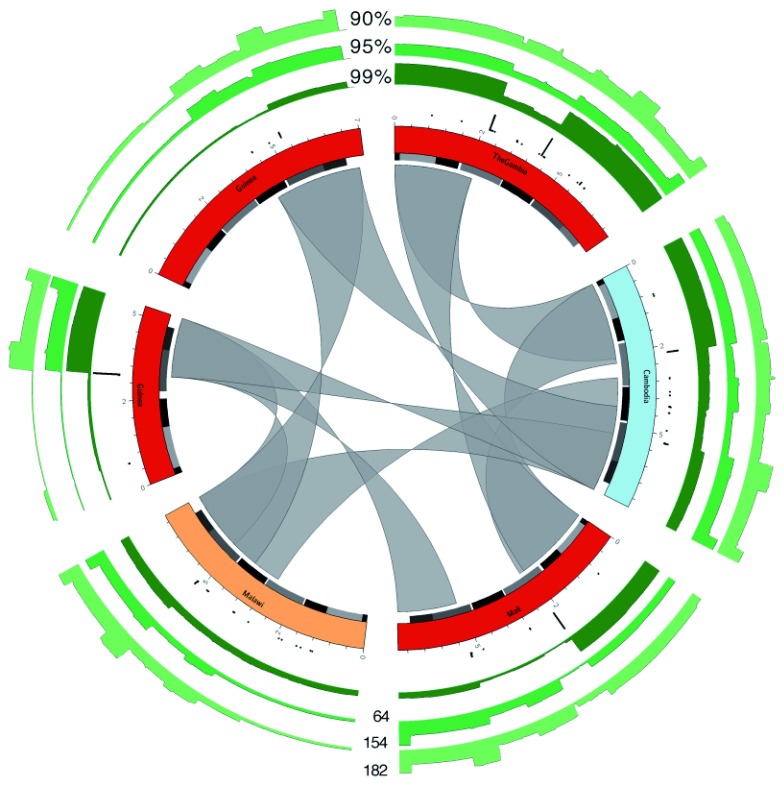
Overview of recombination. Circos plots of six *var* genes, taken from the first 2kb OrthoMCL cluster. Genes are coloured based on the geographic location of the isolate from which they were obtained (using same scheme as Figure 3). Alternating grey and black boxes mark the positions of domains. The inner gray ribbons show similarity between the genes with at least 99% identity and ≥ 2 kb overlap. The black bar plots show frequency of detected recombination events using the normalized *var* gene dataset. The green bar plots show the number of hits over the genes against the normalized dataset, at three different percent identity cutoffs: 99, 95 and 90%. Maximum (y-axis) values are shown against the bar plots at the bottom of the figure.

**Figure 6.**
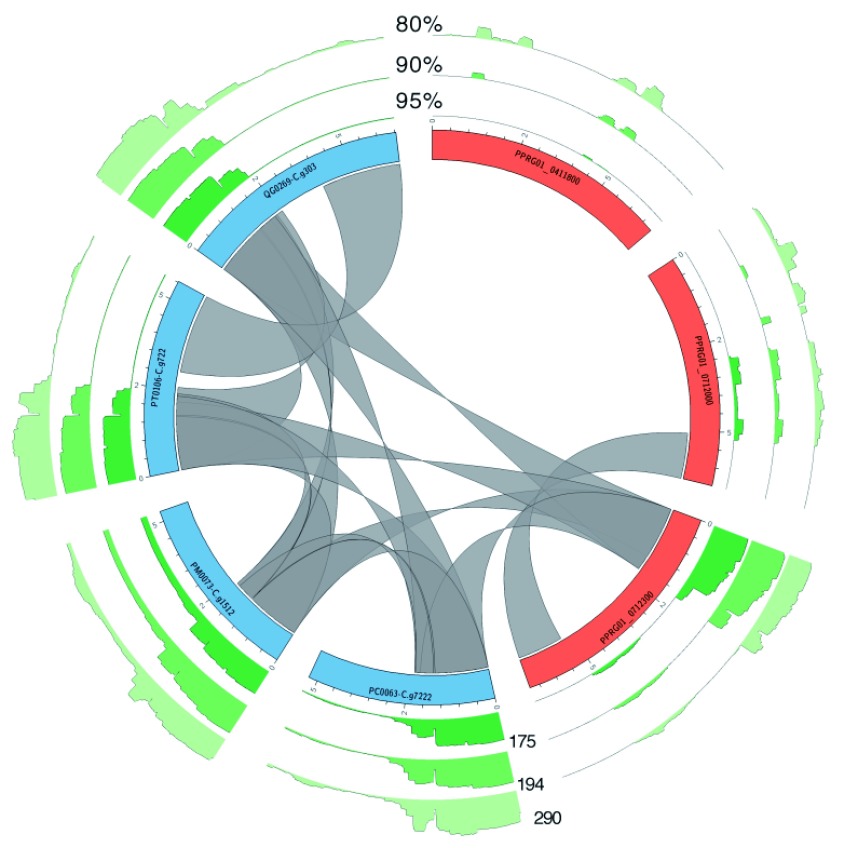
Ancient patterns of recombination within the *Lavernia* sub-genus. Similar to Figure 5, but *P. falciparum var* genes (blue) were selected that hit against a *P. praefalciparum var* gene (orange). The ribbons show matches of ≥ 99% identity 99% and a minimum length of 500 bp.

Definitive evidence of recombination events within the *var* gene repertoire has previously required genetic crosses. However, the global *var* database contains deep recombination history. Having established that long stretches of near-identical sequences represent relatively recently shared sequences, we attempted to investigate recombination by looking for breakpoints in long shared sequence blocks. We called a breakpoint when conserved sequence blocks within the whole dataset of >1kb and >99% identity were interrupted by a second sequence block of >500bp with no hit within the database using our stringent criteria (see Methods, *Extended data:* Table S4) this gave us a total of 42,902 breakpoints in 35,574 *var* genes. Multiple hotspots are apparent from the data ( Figure 5) showing that apparent recombination events within *var* genes were frequently detected but were not random. Within DBL domains, the most frequent events were in DBLδ (41.7% of total) followed by DBLγ (10.4%), DBLβ (8.4%) and DBLα (6.3%). Next most abundant were CIDRα (5.2%) and CIDRβ (2.8%). A large proportion (20.4%) of breakpoints were outside recognised domains ( *Extended data*: Table S5). We also inferred a much deeper recombinational history from the data. For instance, by mapping the totality of the assembled *var* genes against 6 exemplar *var* genes at various levels of sequence-identity ( Figure 5), the identity of the breakpoints was reinforced, with some genes having their entire sequence covered by high-identity hits from within the assembled data. To go back further in time, we repeated this analysis using three extant genes and three genes from *P. praefalciparum* as targets for the mapping ( Figure 6). The deep history of these genes is clear with, in one case, the 5’ third of the gene being highly conserved between current *P. falciparum* and *P. praefalciparum* sequences.

As an alternative approach to looking at *var* gene recombinatorial history, we BLAST-searched our normalised database (with the addition of *var2CSA* sequences) against the 3D7 reference genome, recorded matches (> 1000 bp, > 99% identity) and reported them by UPS type ( Table 3). First, it can be seen that *var2CSA* has a few 1000 bp hits, indicating that despite the relatively conserved nature of this gene enough SNPs must be present to abort the matches. Second, we can see that by this criterion, over 50% of the *var* gene sequences of *Pf*3D7 are unique, highlighting the level of polymorphism that exists. Finally, the upsC type has the most conserved sequences 58%, versus 27% of the other ups types. This class also has more breakpoints, especially in DBLδ ( Table 3), suggesting that the high levels of recombination seen in this ups class and subdomain type may be facilitated by sequence homology ^29^.

**Table 3.** Number of exon-1 sequences of Pf3D7 *var* genes categorised by UPS type. Sequences shared with the normalised dataset with > 99% identity and an overlap ≥1 kb are shown. upsE is the var2CSA gene.

UPS type	*var* genes	Total bases	% genes covered once	breakpoints per *var* gene
UPSE	1	8,004	22.1%	0.0
UPSA	6	50,661	23.6%	1.8
UPSB	37	20,9940	29.8%	1.2
UPSC	13	70,740	57.9%	2.8

Recombination within *var* genes of the progeny of a genetic cross has been associated with nucleic acid secondary structure ^30^. We used exactly the same approach (using RNAfold v2.1.8) to assess the secondary structure across the 100 bp regions centred on our apparent recombination sites. Compared with 100-bp control sequences sampled from all 66 *var* sequences of the PU0134-C sample and the Pf3D7 reference, the free energy distributions look indistinguishable ( *Extended data*: Figure S4) suggesting that the sites we identified are not specifically associated with DNA secondary structure. To determine whether specific sequence signatures define breakpoints, we used MEME to analyse 50bp either side of a random sample of 4,000 detected breakpoints. While there was no single consensus sequence in this analysis, a number of motifs occurred at high frequency and overall had a high GC content (43%) relative to the genome as a whole (19%) and *var* genes in general (36%). One highly significant motif accounted for over ten percent and only nine motifs accounted for nearly fifty percent (2,336 events; *Extended data*: Figure S5).

### Domains and domain cassettes

It is clear from analysis of the Laverania subgenus that the *var* gene family is at least a million years old ^25^. This subgenus is split into two clades (A and B). Within clade B, *P. reichenowi* and *P. praefalciparum* show very similar gene numbers, chromosomal organisation and domain types and domain numbers to *P. falciparum*. Clade A parasites ( *P. gaboni* and *P. adleri*) however show strikingly different CIDR and DBL subdomain content and organisation. Thus, the appearance of what closely resembles the current *var* gene content, organisation and domain structure emerged between 50,000 and 200,000 years ago and has shown no tendency to change significantly since this time. 

The detailed analysis of PfEMP1 proteins to date has been based on data from seven genomes ^13^. In view of the vastly increased volume of data presented here we undertook a reanalysis. Within our expanded dataset, as expected, existing definitions of DBL and CIDR domain types were concordant with existing data in *P. falciparum*. These domain types are also concordant with data from the much more anciently diverged sequences of the group A Laverania parasites, confirming that these sequence arrangements are both ancient and evolutionarily stable. We first carried out an analysis of main domain neighbours within the normalised dataset ( *Extended data:* Figure S6). There are a number of highly enriched domain pairs, DBLα CIDRα as previously reported and DBLζ-DBLε (>99%). Others that occur at high frequency include DBLε-DBLε (56%) and DBLβ –DBLγ (64%).

In view of the data using existing subdomain definitions that relates expression of particular domain cassettes with parasite phenotypes and disease severity we undertook an analysis of domain cassettes within our data. The complete analysis is presented in *Extended data*: Table S6 in which combinations of at least three subdomains that occur at least 10 times are shown. We found a total of ~86,000 triplets representing 1,567 different combinations that clearly included the previously defined domain cassettes. Four novel domain cassettes occurred >1,000 times and comprised ~7% of the total. We could not find a significant association of domain types with geographical regions.

**Figure 7.**
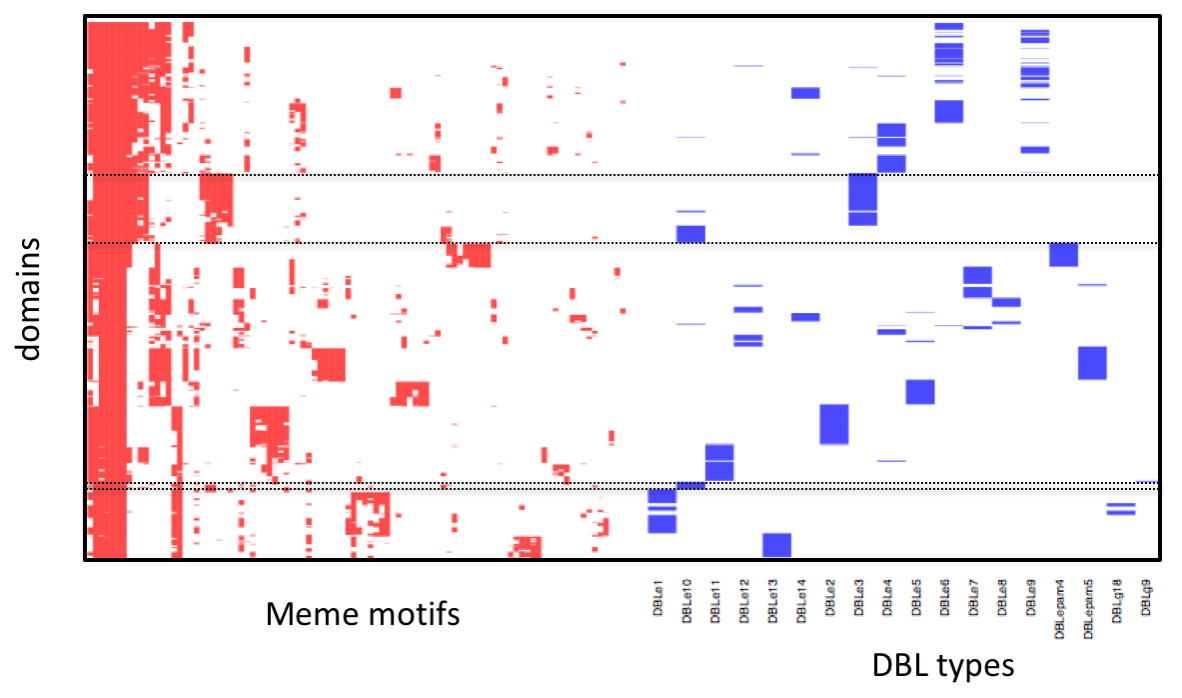
Visualisation of motifs in DBLε domains. Presence of individual MEME motifs (columns) in each DBLε domain (row) is shown (red). For each domain, annotation of subdomains, according to the VarDom server, is also shown (blue). The top dotted box shows the similarities between DBLε4 and DBLε10 and thus that they should be combined into a single subdomain. The bottom dotted box shows that a small sample of DBLε10 clusters with either DBLε1 or 11.

Sub-domain definitions are currently based on trees drawn from multiple alignments of sequences of seven genomes. However, tree-based approaches are not ideal for showing relationships between highly recombinogenic sequences and have produced some anomalous results ^31^. As an alternative, we used MEME to identify 256 six-nucleotide motifs within each domain or domain type and then clustered these by their co-occurrence within a single sequence. As an example of the utility of this approach we used it to classify the DBL and CIDR domains from the *Laverania* ^25^ ( *Extended data*: Figure S7). The robust identification in the *Laverania* of the main domains from *P. falciparum* reinforces the conclusion that the main domain architecture within the *Laverania* has been stable over long evolutionary time scales. We next applied the same approach to subdomains of *P. falciparum*. For example, Figure 7 shows the clustering of 1,000 DBL epsilon domains projected onto existing subdomain classifications. It is clear that there are a number of anomalies. For example ( Figure 7, top box), DBLε10 and DBLε4 appear to be a single subdomain and a small portion of DBLε10 clusters with DBLε1 and DBLε11 ( Figure 7, bottom box). By contrast, when we carry out the same set of analyses on DBLα sequences, the clustering ( *Extended data*: Figure S8) clearly distinguishes between the major subclasses (0, 1 and 2) but does not reveal the same degree of substructure. The MEME analysis therefore suggests much more heterogeneity in the data making it difficult to assign robust sub-domains in this case.

**Figure 8.**
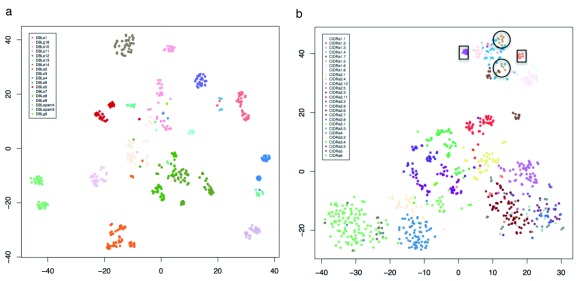
Meme motifs DBLε and CIDRα domains. Matrix of MEME abundance (from Figure 7) visualized using t-SNE plots for ( **a**) DBLε and ( **b**) CIDRα.

Another method of viewing the high dimensionality of this data is a nonlinear t-SNE projection in two dimensions ( Figure 8). For DBLε the tight clustering of the data into groups is again apparent ( Figure 8a). Since the main goal of such sub-classifications is to relate sequence to phenotype, we looked closely at CIDRα1 sequences, the majority of which are known to bind to EPCR and whose expression has frequently been linked to severe malaria. When we do this, we find a clear discrete clustering ( Figure 8b, boxed) of subtypes CIDRα1.2 and 1.3 that are the only two that show no binding to EPCR. The other subdomain that shows variable EPCR binding is CIDRα1.5 and this splits into two groups (circled). The rest of the projection clearly shows that while some of the data cluster consistently with current subdomain definitions, many do not, suggesting that the current CIDR subdomain definitions are not robust when analysed in this manner. The same projections for other DBL and CIDR domains are presented in *Extended data*: Figures S9-S11. The general conclusion from these figures is that while some existing subdomain definitions look robust to this analysis many do not. The most clearly resolved clusters can be seen in DBLε and DBLζ. DBLε is most closely related to the original and likely the most ancient DBL domain since it is found in many species and is present in proteins involved in red cell invasion. DBLζ is most abundant in the clade A *Laverania* that diverged from current *P. falciparum* populations around 1 million years ago ^25^. This suggests that maybe because of their age, the subdomains in these classes may have functionally diverged (for example DBLε has diverged from other DBL domain types to bind IgM ^32^), such that recombination between them is not favoured. In contrast, in the majority of other cases, functional diversification is not yet complete such that limited recombination is still going on. However, it should be noted that because the granularity of the output is dependent on the length of the MEME motifs included in the analysis, the parameter space is too large for us to explore all possible outputs but suggests that once a strong phenotype can be associated with a given domain type then this approach may be valuable in tying sequence variation to phenotype.

**Figure 9.**
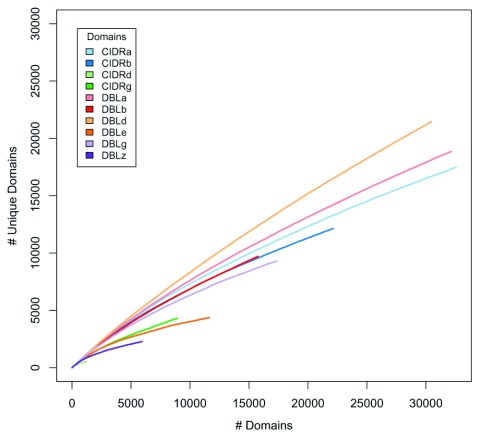
Diversity projection of domains. Accumulation plots of the number of domains versus the number of unique domains. Only hits with ≥ 99% amino acid sequence identity over their full lengths included.

**Figure 10.**
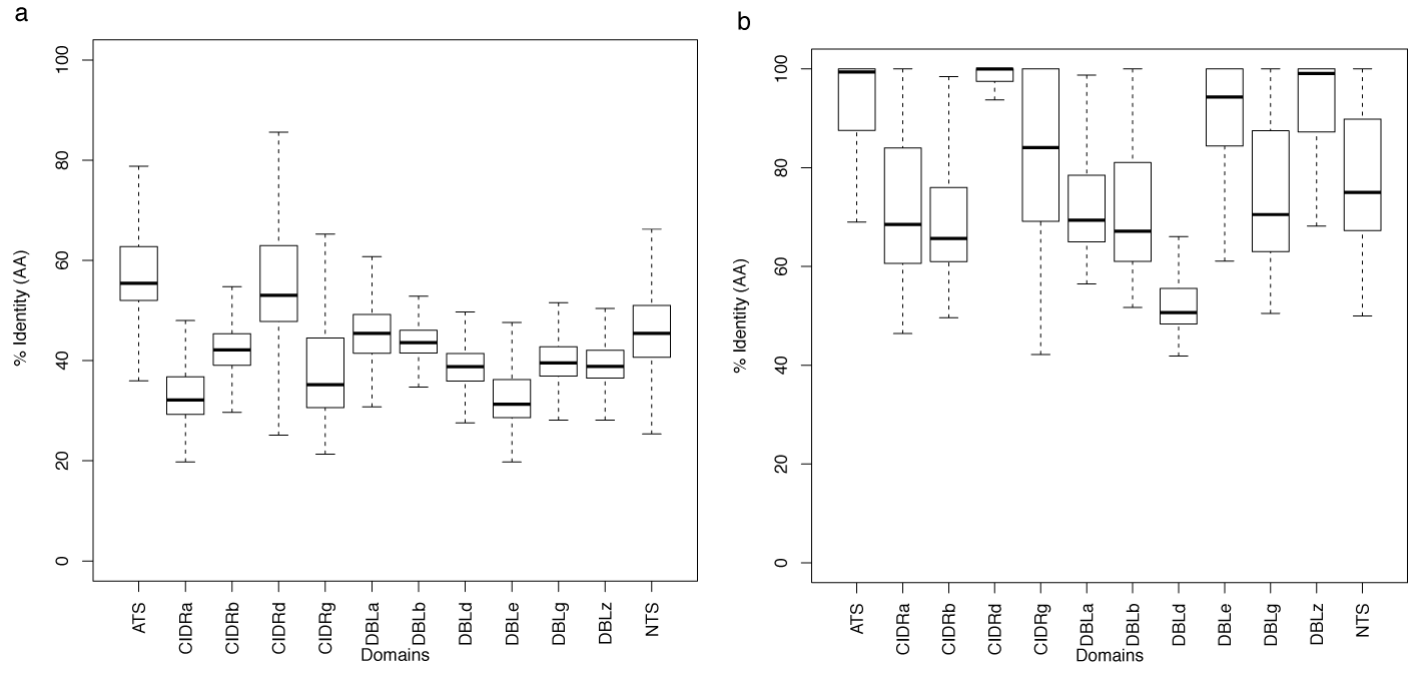
Diversity in domains. Boxplots showing sequence identity for 1,000 randomly selected sequences from each domain type based on an all-against-all BLAST analysis. In ( **a**) all results are shown, in ( **b**) the sequence matches for the top hits are shown.

As an alternative approach to examining the way in which the main domain types have evolved and continue to do so we examined their relative diversity in two ways. First, we plotted accumulation curves of the number of new sequences with increasing sample size to show that the level of world-wide sequence diversity varied significantly with domain type ( Figure 9). Most diverse were CIDRα, DBLα and DBLδ while the least diversity was seen in DBLζ and DBLε (consistent with the results above). Next, we took each domain individually and carried out an all against all BLAST. The results were then visualised as box-plots either of the overall diversity or as the %identity of the top BLAST hit ( Figure 10a). The mean BLAST similarity varied from 30–80% with the most conserved domains being those that constitute *var2CSA* and the most diverse being DBLε. These values are similar to those described by 13. However, when the identity of the top hit was plotted ( Figure 10b) a different picture emerges with DBLε having the highest identity. This is again consistent with the results above because of the well separated subgroups: within the subgroups identity is high but the groups are very diverse from each other.

## Discussion

While there have been a large number of studies that have attempted to analyse the local or regional distribution of *var* gene sequences, these have to date mainly been limited to amplicons containing DBLα sequences, eg: ^4– 9^. Some smaller studies have included full length sequences but to date these have not involved sampling across the world. This therefore represents the first large scale attempt to catalogue the worldwide distribution of *var* gene sequences. We draw a number of conclusions from this study. First there is an unexpected level of *var* gene sequence sharing across the globe that falls into three main categories:

(1) A very high degree of full-length sequence sharing in South East Asian isolates that is likely due to recent selection for parasites resistant to artemisinin;
(2) A small proportion of sequence sharing that appears to originate from drug induced selective sweeps;
(3) Sequence sharing that appears to have resulted from the recent intercontinental movement of parasites

In the case of (1) we find that the analysis of the *var* gene content alone is sufficient to partition the population into the same subgroups as well-established SNP-based population genomics approaches ^33^ and if we increase the resolution by determining the number of isolates that share >50% of their *var* gene repertoire it becomes clear that there has been an almost clonal expansion of certain genotypes in some areas of this region ( *Extended data*: Figure S3). By adding the date of collection to this analysis we can confirm that selection is still continuing, by the implementation of artemisinin combination therapy where resistance to the partner drugs is already appearing ( *Extended data*: Figure S3). Thus, because *var* genes represent markers across the genome they can be used independently of SNP analysis to study recent selection events.

Second and somewhat surprisingly we can see in the distribution of *var* gene sequence the remnants of much older selective events involving chloroquine and anti-folate drugs on chromosome 7, 4 and 8. Why this signature has been retained in some isolates and not others is not entirely clear. In the case of chromosome 7, those isolates that have retained six identical genes in the internal cluster are characterised by the presence of one (the fifth *var* gene) on the opposite strand that may have restricted recombination. Nevertheless, the fact that this event is present in 22 isolates suggests that there must have been some restriction on recombination since selection. Why the signature has presumably been retained on chromosomes 4 and 8 in some isolates only is unclear. With regard to chromosome 6, a local selective sweep of unknown cause has been reported in West Africa ^28^ and this may well be the same signature that we are detecting. The origin of the *var* gene sharing on chromosome 12 is at present unclear. Since we only had 12 PacBio genomes with which to search our database, it is likely that a much higher proportion of these shared genes would be revealed as remnants of selective sweeps if we were to search a larger number.

Third, there is an additional transcontinental level of *var* gene sharing that does not appear to be chromosome enriched and therefore does not show evidence of a selective sweep. Our analysis suggests (because the length of sequence shared is related to the percent identity) that these simply represent recent recombination events that have resulted from parasite movement as a result of international travel. This observation reinforces the worry that there is background level of global parasite movement that may have consequences for the spread of drug resistance. These three different types of sharing are clearly evident in Figure 3b and c.

By analysing our database as a whole we were able to identify likely recombination events involving breaks of two blocks of identical sequence. These events are not random but instead are most common in DBLδ domains (41.7%) and outside of DBL domains (20.4%). It is encouraging that the degree of domain structure revealed in the MEME t-SNE plots seems inversely related to the amount of recombination observed reinforcing the conclusion that some domain types are now relatively stable while others are still evolving. If we concentrate on those SE Asian isolates that share a high proportion of their *var* gene repertoire we can readily detect recent recombination events that appear to be more frequent in genes of the UpsC type ( *Extended data*: Figure S3). In contrast with a previous report ^30^ we do not find evidence of nucleic acid secondary structure associated with the breakpoints but do find a limited number of MEME motifs with a high GC content that account for ~50% of the breakpoints.

With regard to the unusual *var* genes that are relatively conserved, we reinforce the observation that *var1CSA* has two alleles that first separated within the *Laverania* infecting apes ^25^. Within both major subtypes there has been a degree of diversification ( *Extended data*: Figure S1) but no evidence of recombination suggesting that they are being maintained within the population by balancing selection. However, since the function of this gene is unknown, the source of this selection cannot yet be identified.

*var2CSA* sequences have been analysed elsewhere ^26^, however, unexpected in our data was the presence of a number of highly elongated sequences extended by large numbers of DBLε domains and a region of ~6 kb in some isolates that is identical to a region of the gene from *P. praefalciparum* ( *Extended data*: Figure S2). While the function of this gene in placental cytoadherence is well established it has also been reported to be involved as a central switching intermediate in antigenic variation ^34^. Some DBLε sequences are known to bind IgM but whether this is pathologically relevant, what role the extended sequences might play and why some have been maintained since the *P. praefalciparum* split are unknown. *var3* sequences show high sequence homology (~98%), a degree of copy number variation and some rare variants containing an alternative NTS sequence and a DBLα domain.

The cataloguing of different domain and subdomain types has been important in *var* gene research since it has been established that certain domain and subdomain types as well as combinations of them (known as domain cassettes, DC) are involved in binding to specific host receptors and associated with severe disease. Whether these DCs are functional units or not is however unclear since in the best studied case of DC8 and DC13 binding to EPCR, binding appears to be mediated by CIDRα alone ^35^. While we had no clinical phenotype data here and have analysed genomic sequence rather than transcript data, our re-analysis nevertheless revealed some novel interpretations. While some domain types (particularly DBLε and DBLζ) show robust clustering into set of clear subdomains, the clustering of other DBL domains is less clear. The former two are likely the most ancient as they are the most numerous in clade A *Laverania*. In addition, DBLε is closest phylogenetically to the DBL type involved in other functions such as invasion, present across the genus and therefore likely to be ancient. They therefore appear to have segregated into what are presumably functional subtypes that now show little propensity for recombination to split them. In the case of the other types, we know that they have been long established since they are present in their present form in clade B *Laverania.* However, the inability to cluster them into unambiguous subtypes in most cases shows that a significant degree of recombination between them is still occurring. Indeed, our conclusion that this is correct is strengthened by the fact that the domain in which we found the most recombination events (DBLδ) coincides with the MEME analysis in which we found the least structure. The fact that they have not segregated into such distinct classes suggests that there is an ongoing process of optimisation possibly driven by host polymorphism in the host proteins with which they interact.

## Conclusion

We have assembled essentially complete repertoires of *var* genes from global isolates of *Plasmodium falciparum*. This new resource enables key aspects of the evolution of this important multi-gene family to be unravelled. The repertoire has ancient routes since the number, domain content and genomic organisation have been established for more than 500,000 years ^25^. Within that time frame, some domain types appear to have evolved to a relatively stable state that is structured but still polymorphic and that we hypothesise is related to the optimisation of their functions. Most domain types however appear to be still evolving as evidenced by the detection of recombination breakpoints and by the absence of defined structure in the t-SNE analysis. These breakpoints are characterised by a limited number of sequence motifs. Thus, while the evolutionary fitness of the family as a whole must be very high because of its longevity, optimisation is still occurring presumably driven either by function or by immune pressure.

## Methods

### Samples

Illumina sequence data were obtained from the MalariaGEN *Plasmodium falciparum* Community Project and the Pf3k project ^36, 37^. In addition, we included 12 unpublished Kenyan samples (PFKE01 - PFKE12) that will be described in more detail elsewhere. All samples (except the latter 12) were obtained directly from patients and sequenced using Illumina technology with read lengths of 75 or 100 base pairs.

### Pipeline for assembly and annotation of var contigs

Sequence data for each sample were mapped against the reference genome (Pf3D7 version 3 from GeneDB) ^38^. From the resulting mapping file (in BAM format), reads were extracted that either did not map to the reference genome or that mapped to *var* coding sequences, plus 500 bases up- and down-stream. Sequences were assembled and annotated using bespoke Perl and BASH scripts that executed the following steps: (1) Reads were assembled *de novo* using Masurca ^39^. (2) Overlaps (> 200 bp) between contigs were determined using MegaBLAST (parameters: -W 40 -F F -m 8 -e 1e-80) and contigs were excluded if they overlapped for more than 90% of their lengths, with identity ≥ 99%. (3) Illumina sequencing reads were mapped using SMALT (version 0.7.4, default parameters) to the full set of contigs. Contigs were merged with a Perl script ( findoverlaps_ver3.pl, https://github.com/ThomasDOtto/IPA/) if the overlap region was > 500 bp and if the coverage of mapped reads across the overlap region was 50% of the median coverage value, determined from the whole assembly. (4) Contigs were joined into larger scaffolds using SSPACE ^40^ (version 2.0, parameters -n 31 -x 0 -k 10). (5) Gaps were closed between contigs using IMAGE ^41^, for two iterations with k-mers of 71, and minimum mapping scores of 65. (6) Small errors were corrected with iCORN ^21^ (version 2) for two iterations from reads aligned using Bowtie2. (7) Miss-assemblies were detected and broken based on aligned paired reads, using REAPR (version 0.7.4 ^42^, parameter -a). (8) Coding sequences were identified in each contig using Augustus ^43^ (version 2.7) trained on the *var* genes of *P. falciparum* 3D7 version 3 and *P. reichenowi* PrCDC ^18^. (9) The identified coding sequences were annotated with functional descriptions based on BLASTp, and Perl scripts were used to write out the nucleotide and amino acid sequences. (10) Estimation of coverage was performed by mapping the Illumina reads back against the extracted *var* coding sequences with SMALT (as above), read depth was counted as normalised to FPKM values.

### Evaluation of assemblies

During development, the assembly algorithm was tested using paired reads (75bp, 150bp and 250bp) produced from the Pf3D7 reference (DNA was from the same stock as the reference genome project ), with a fragment size of ~500 bp, slightly longer than the Pf3K samples (mean 380 bp).

For quality-control purposes, whole-genome assemblies were used that were produced using single molecule real time sequencing (Pacific Biosciences) as part of the Pf3k project ^16^.

A “reference set” of *var* genes, from both Pf3D7 and 15 Pf3k reference genomes, was used to evaluate the *var* genes assemblies (hereafter termed “Illumina set”). The DNA used for the Pf3k reference genomes was also sequenced on the Illumina HiSeq platform, using the same protocol as for the field samples (100bp paired reads ^44^). The Pf3D7 DNA was from the same stock as the reference genome project and was sequenced on a MiSeq with 250 bp paired reads from a PCR-free library. 

All predicted *var* genes ≥ 3kb from the Illumina set were compared, by BLAST-searching, against the coding sequences and raw assembly (output of HGAP ^45^) of the reference set. Matches identity of 99.5% identity, covering > 95% of the shortest sequence were counted. LARSFADIG is a conserved amino acid sequence known to be present in the DBLα domain of *var* genes and its presence was used to crudely estimate the number of *var* genes in the uncorrected and final Illumina sets. Two samples from the reference set were known to contain mixed infections. However, these samples did not contain the full-expected number of *var* genes; several were present in a partially assembled form on contigs < 5kb that had been excluded from the Pf3k reference genome pipeline.

Pacific Biosciences reads were used to validate the contigs of the Illumina set for each isolate. First filtered raw reads were chunked in 500 bp pieces and mapped independently using BWA-MEM ^46^ (parameters: -x pacbio -t 16 -a -S -P). Each 500 bp sub-read was mapped as often as possible (parameter: –a) and discontinuities in the mapping of sub-reads were checked manually using BAMview ^47^. For a correctly assembled *var* gene, the sub-reads should map continuously over the full length of the var gene. Errors were defined, if the first hit of a gene in the Illumina set had a non-mapping region > 200 bp, and the long mapping or validation approach confirmed the error.

The completeness of each assembly was calculated as the sum of all matches (> 500 bp overlap > 99.5% identity) between the genes (> 500 bp) of the Illumina set and the genes (> 3 kb) of the reference set, divided by the total length of all *var* genes > 3 kb in the reference set.

To establish the error rate, we divided the number of incorrect *var* genes from the 16 Illumina *var* gene assemblies by the total number of *var* genes > 3kb.

For the further analysis we used 12 PacBio Reference genomes and Pf3D7.

### Normalized dataset

As the number of samples per country varied across the whole dataset, a dataset comprising assemblies from 60 isolates, from each of the 12 countries, was extracted. Where possible, samples were selected with close to the expected number of LARSFADIG motifs for a single parasite genotype (between 45 and 90 LARSFADIG sequences). Where this was not possible, the number of LARSFADIG sequences were increased. If still not enough samples could be retrieved, the lower limit was decreased. As we were interested in the variable first exon, rather than the more conserved exon 2, the 3’ end of each *var* gene was excluded if it matched (BLASTx -m 8 -e 1e-30 -b 5 -v 5) the exon 2 amino acid sequences from Pf3D7 or *P. reichenowi* PrCDC.

Conserved *var* sequences, like *var1CSA*, *var2CSA* and *var3* (< 4kb), see below, were also excluded. The remaining *var* genes were compared with a MegaBLAST (-F F -e1-80). High sharing between several Gambian samples was observed, possibly due to repeated infections of the same vector in low endemic regions ^48^. In four Kenyan samples (PC0016-C, PC0025-C, PC0080-C and PC0083-C), identical hits to PfDd2 were identified and these samples were also excluded.

In total, a normalized set of 714 genomes from 12 countries was generated that comprised 39,119 sequences ≥ 3kb from exon I. This dataset was used for most of the analysis, including general sharing, recombination, duplications. A further data set was created for use in Figure 4 that comprised additional sequences from Cambodia and Thailand, where they contained at least 40 LARSFADIG sequences and if they had a least 20 shared *var* genes to the Cambodian samples.

### *var1CSA, var2CSA, var3* analysis

To identify *var2CSA* copies across the data set, Pf3D7 *var2CSA* (Pf3D7_1200600) was BLAST-searched against the normalized *var* gene set. Contigs with an identity >95% were considered to be *var2CSA* candidates. Contigs > 3kb were further verified by checking for the presence of known *var2CSA*-specific domains (DBLpam1-3 and CIDRpam).

Using the three *var3* genes from Pf3D7 as query sequences (PF3D7_0100300, PF3D7_0600400 and PF3D7_0937600), copies of *var3* were detected in the global set using BLASTn with a minimum of 95% identity, an alignment length of ≥ 1kb, and a maximum gene length of 4kb.

The detection of *var1CSA* was more difficult due to its similarity with other *var* genes. However, the first 3.2 kb, from the 5’ end, is particularly conserved and this region was used to discriminate *var1CSA* from other *var* genes. A Maximum Likelihood tree was constructed from 5’ 3.2 kb regions of *var1CSA.* Alignments were generated with MUSCLE ^49^ and cleaned in seaview ^50^ first manually, then GBLOCKS ^51^, default parameters). Trees were drawn with PHyML ^52^. Alignments were visualized in JalView ^53^. To analyse sequence ** variation across the lengths of *var1CSA*, copies were aligned with ≥ 80% overlap with BWA-MEM against either of the two *var1CSA* types, and variants called with SAMtools Pileup and BCFtools.

### Domain analysis

Domains were first defined in the *Plasmodium falciparum* genome as part of the genome project ^2^. The domains were refined subsequently using *var* genes from seven lab-strains ^13^ and are accessible through the VarDom server. From there HMMer models ^54^ were obtained. Amongst those HMMer models, is the “duffy binding like” domain defined by Pfam ^55^. The HMMer models for the subdomains were built from the amino acid sequences extracted from the VarDom server. In all *var* genes longer than 3kb, the positions that encoded domains were identified using hmmscan (parameters: --noali -E 1e-6 --domE 1e-6 ^54^, and the HMMer models from the VarDom server. Domains were assigned to the sequence, first by the highest score and ensuring that domain coordinates did not overlap by more than 10bp. The output of HMMer was parsed into a GFF file and a list of domains per gene. This enabled the domain order and distance between domains to be determined.

Domains from a further six *Plasmodium* spp from the *Laverania* subgenus were obtained in a previously published dataset ^25^.

### Domain evolution

The domain diversity was investigated by searching for conserved motifs. MEME motifs ^56^, of 8–15, four or six amino acids were identified, rather than the perfect matches that have previously been reported ^13^. Basically, the programme meme ^56^ search for the 96 or 256 most abundant motifs amongst CIDR or DBL domains from the seven Laverania genomes and 1000 random *P. falciparum* domains from each of the 18 domain types (ATS, CIDRα, CIDRβ, CIDRδ, CIDRγ, CIDRpam, DBLα, DBLβ, DBLδ, DBLε, DBLγ, DBLpam1, DBLpam2, DBLpam3, DBLζ, Duffy_binding_like, NTS, NTSpam). For visualisation, the domains for each given gene were parsed from the meme.txt output file into a binary matrix. Each row represents a different *var* domain and the column show the different meme motifs. A matrix entry is set to 1 if a meme motifs occurs in a domain. Using the heatmap2 function (ggplot2 package ^57^) and the average hierarchical clustering method in R ^58^, domains were visualized per sequence as a clustered distance matrix. In some cases, metadata like domain type or species were attributed as a barcode. To generate the t-SNE plots the R function Rtsne (parameter: perplexity=30, max_iter = 2500) from the Rtsne library was used.

### Recombination analysis

Breakpoints were obtained from successive filtering of Blast comparisons. First all the exon1 sequences of the normalized dataset were compared with themselves using megablast (-F F).

Alignment breaks as defined by BLAST matches are breakpoints if following criteria are met: high quality match (>1kb and >99% identity) between query and subjects, the sequence match does not span a gap, and the sequence of query and subject extends at least 200bp further. Also, the query and the subject cannot have a subsequent hit (identity > 95%) within 500bp of the sequence match. The potential presence of stable secondary structures was investigated by calculating free energy using RNAfold ^59^. From each predicted break points, 50 bp sequences up- and downstream were passed to RNAfold (version 2.1.8 ,parameters --paramFile=dna_mathews1999.par --noGU --noLP --noPS --gquad --noTetra), and minimum energy was taken, as described in 30. As a control, the free energy of 100 bp sequences of all 66 exon1 sequences of PU0134-C (66 in total) were generated and the minimum energy was taken for each 100bp sequence. Histograms were plotted in R. This was repeated on the *var* genes of the Pf3D7 reference to generate a figure ( *Extended data*: Figure S4).

### Duplication analysis

To estimate duplication within a sample, the calculated RPKM of genes longer than 3kb were used. First multiple infections were excluded (as samples with more than 90 LARSFADIG motifs were excluded in the normalised dataset). If a *var* gene had an RPKM > 1.85 times of the median, the gene was considered duplicated. If the RPKM was between 1.35 and 1.85 of the median, the var genes was considered partially duplicated. Those values were manually checked in BAMview ^47^.

### Analysis of var sharing between isolates

OrthoMCL ^60^ was used with default parameters to cluster *var* sequences. The BLAST input was generated with MegaBLAST -F F and the identity and overlap were filtered with awk to generate clusters with different specific cutoffs.

Networks isolated linked by shared *var* genes were generated with Gephi ^61^ clustering performed through the Fruchterman algorithm ^62^.

### Analysis of var genes from Southeast Asia

The Southeast Asia dataset was visualized with Gephi, see above. To colour the nodes, the position of the mutation in the Kelch13 gene (PF3D7_1343700) was used. It was called from the BAM files using mpileup (SAMtools ^63^ and BCFtools. Metadata for time and location were used from ftp://ngs.sanger.ac.uk/production/pf3k/release_5/pf3k_release_5_metadata_20170804.txt.gz.

### Ethics approval and consent to participate

All samples were previously published and had ethical consent.

## Data availability

### Underlying data

Zenodo: Extended data for "Evolutionary analysis of the most polymorphic gene family in falciparum malaria", http://doi.org/10.5281/zenodo.3549732 ^64^.

This project contains the following underlying data:

- Additional File 1: Table S7: Overview of all assembled samples with >10 LARSFARDIG motifs. Key metadata are shown, along with the number of LARSFADIG motifs and *var* genes at different length cut-offs available.

The accession numbers of the raw data from 12 previously unreleased Kenyan samples are in Additional file 1: Table S7. All other accession numbers can be found from the MalariaGEN *P. falciparum* Community Project Pf6 release at https://www.malariagen.net/resource/26 or the Pf3k release at ftp://ngs.sanger.ac.uk/production/pf3k/release_5/pf3k_release_5_metadata_20170804.txt.gz

Code used to generate the *var* genes: https://github.com/ThomasDOtto/varDB.

Archived code as at time of publications: https://doi.org/10.5281/zenodo.3549770 ^65^

License: GNU General Public License v3.0

*var* gene sequences and the domain information can be found on the github and also on ftp:// ftp://ftp.sanger.ac.uk/pub/project/pathogens/Plasmodium/falciparum/PF3K/varDB/.

Data can also be accessed by BLAST-searching from https://www.sanger.ac.uk/action/BLAST

### Extended data

Zenodo: Extended data for "Evolutionary analysis of the most polymorphic gene family in falciparum malaria", http://doi.org/10.5281/zenodo.3549732 ^64^.

This project contains the following extended data:

- Additional File 1: Tables S1-S6.
- Additional File 2: Figures S1-S11.

Data are available under the terms of the Creative Commons Attribution 4.0 International license (CC-BY 4.0).

## Acknowledgements

This study used data from the MalariaGEN *Plasmodium falciparum* Community Project. Genome sequencing was done by the Wellcome Sanger Institute; sample collections were coordinated by the MalariaGEN Resource Centre. We thank the staff of Wellcome Sanger Institute Sample Logistics, Sequencing, and Informatics facilities for their contribution. We thank all patients and collaborators to the MalariaGEN *Plasmodium falciparum* Community Project. We would also like to thank the Pf3k consortium and collaborators for the access to the data. We thank Pete Bull and Abdirahman Abdi for the 12 Kenyan samples.
