# Supplementary material for: Evolutionary analysis of the most polymorphic gene family in falciparum malaria
Source: Wellcome Open Res. 2019 Dec 3;4:193. [Version 1] doi: 10.12688/wellcomeopenres.15590.1 (PMC7001760; doi:10.12688/wellcomeopenres.15590.1)
Supplement: Peer review history

# Peer review: Reviewer response for version 1

**DOI:** 10.21956/wellcomeopenres.17073.r37595

**Published:** 2020-02-04

**Authors:** Daniel B. Larremore

**Affiliations:** 1 Department of Computer Science, University of Colorado Boulder, Boulder, CO, USA; 2 BioFrontiers Institute, University of Colorado Boulder, Boulder, CO, USA

This is a review of "Evolutionary analysis of the most polymorphic gene family in falciparum malaria" by Otto *et al.* The other reviewers have summarized the paper in their comments, so I will not rehash their summaries.

 In short, this highly anticipated analysis represents a fantastic amount of work, assembling 2400 var repertoires from a globe-spanning set of *P. falciparum* genomes. The analysis is thorough, well documented, interesting, and revealing. It is written at a level that is easily readable by the var community while also being interesting (I think) to the wider community. 

 Still, I have a few suggestions and comments which I hope will increase the impact and improve the readability of the manuscript. 

**Limitations:**

- I found this paper to be really strong, and I have no doubt that it will inspire substantial downstream research from many groups. To that end, I'd like to encourage the authors to include, at the end of the discussion, some thoughts on the limitations of the study and its interpretations of the data. 

**Multiple infections:**

- I am mildly concerned about the impact of the MOI>1 samples. If the goal of imposing the 90 LARSFADIG threshold is to avoid MOI>1, but the reason that the African isolates have higher average numbers of LARSFADIGS is due to higher MOIs, this makes me wonder if the cutoff is too permissive. It seems self-contradictory to say that a cutoff was imposed to limit MOI, but that higher average counts were because the cutoff let higher MOI samples through. At the same time, there doesn't seem to be anything to do other than to possibly vary the cutoff, which (I'm guessing) would result in a statement like "we varied the cutoff and the results do not substantially change."  
- However, this came up again during the Gene Duplication section on Page 8. What fraction of the gene duplications or partial duplications could be based on multiple-infection samples? It seems like some of the observed duplications here could be attributable to both (1) actual partial or complete duplication and (2) multiple infection. I don't know what the right null model would be for the scaling of duplications with number of vars in confirmed MOI=1 infections, but it would be interesting to see the extent to which larger repertoires were predictive of these partial and complete duplication observations. 
- Perhaps relatedly, or perhaps not, the Evaluation of Assemblies section of the Methods refers to "Two samples from the reference set" which contained mixed infections. I didn't understand the implications of that paragraph for MOI and QC more broadly. 

**Be careful with tSNE:**

- tSNE is fantastic but lacks interpretability in ways that are familiar from PCA or embedding methods. I won't recapitulate the issues with it, but I would like the authors to check that other tSNE perplexity values don't change their visually appealing results and interpretations. This page recaps issues with tSNE in a visually compelling set of examples. https://distill.pub/2016/misread-tsne

**Minor things:**

- The copy number variation of var3, noted on Page 5, struck me as interesting. Is there anything interesting that can be said of the copy number distribution? 
- Figure 3b is difficult to make sense of, but I wondered what it would look like as a heatmap of the network's weighted adjacency matrix, organized by region? I'm guessing that there's plenty of structure, but the sheer number of edges in the network obscure it. 
- On Page 8 it says, "The bottleneck that produced these different populations probably occurred around 20 years ago." Is this an actual probabilistic statement or Occam's Razor reasoning based on the patterns in Figure 4? I got thrown off by "probably," in other words.
- On Page 8, in the Recombination section, it says that definitive evidence has previously required genetic crosses. Is the Claessens *et al.* (2014 ^1^) work not definitive? (This is an honest question – it is not meant to be rhetorical.)
- On Page 10, in the discussion of Domain Cassettes, you note some enriched domain pairs (DBLz-DBLe). First, what do the parenthetical 99%, 56%, and 64% mean, exactly? Second, are there also domain pairs that are obviously precluded, or domains that are mutually exclusive?
- Figure 7 was a mystery to me. My general impression is that the MEME motifs - the horizontal axis label has inconsistent capitalization of MEME, by the way - support the DBL types but not the subtypes. In other words, the argument is that the previously constructed subtypes were overfitting the data, and dividing domains into substructures that aren't truly meaningful. That's fine, but the figure itself remains confusing. What are all the dotted lines? Which ones do match? Can the font sizes be increased, too?
- In the Discussion on Page 13, you write that "These events are not random but instead..." and then there are parenthetical percentages. What do these percentages mean? 

**Trivial/Typographical suggestions:**

- Abstract: periods at the ends of the first two paragraphs?
- Results: "from approximately 2400" -> 2398. No need for rounding in line one of the results. 
- Figure 1: What are the vertical axes? Annotations are hard to read. Could colors that are higher contrast than orange/brown be used, by chance?
- Figure 2: Are higher resolution (or vector) formats available? 
- Page 4, last paragraph: "epsilon" is spelled out, inconsistent with the rest of the text. 
- Page 7, left column: The sentence that contains "our PacBio assemblies were BLAST-searched against our database" was confusing to me. Something about the wording and the "our"s made me question whether I understood exactly what was meant by our PacBio assemblies and our database. 
- Same as above. "In the former case" confused me, given the complexity of the previous list. Could you be explicit about the case? 
- Same as above. "in a subset of isolates (22)".  I printed the paper to read it and couldn't tell if this was a parenthetical reference or a number of isolates. 
- Page 7, right column: "clustered by the Kelch mutation" - I think this is not exactly correct. The nodes are *colored* by their Kelch mutations, but visually clustered by their var repertoire overlap through the network layout, right? 
- Page 8: "an isolate specific analysis" -> "an isolate-specific analysis".
- Figure 5 is very interesting, but I'd love to see it larger, like Figure 6 is. 
- Page 9, left column: "First, it can be seen...must be present to abort the matches." I didn't understand this sentence. Could it be clarified?
- Figure 8 has annotations that are described in the text, but not in the figure caption - but the figure precedes that main-text description.
- Figure 9: Can you just annotate the curves directly? I bounced back and forth between the legend and curve and had to just draw the annotations on the paper with a pen in the end. 
- Page 14: "Miss-assemblies" -> Misassemblies?

I confirm that I have read this submission and believe that I have an appropriate level of expertise to confirm that it is of an acceptable scientific standard, however I have significant reservations, as outlined above.

## References

[1] : Generation of antigenic diversity in Plasmodium falciparum by structured rearrangement of Var genes during mitosis. *PLoS Genet*.2014;10(12) : 10.1371/journal.pgen.1004812 e1004812 10.1371/journal.pgen.1004812 25521112PMC4270465

# Peer review: Reviewer response for version 1

**DOI:** 10.21956/wellcomeopenres.17073.r37260

**Published:** 2019-12-19

**Authors:** Thomas Lavstsen

**Affiliations:** 1 Centre for Medical Parasitology, Department of Immunology and Microbiology (ISIM), Faculty of Health and Medical Sciences, University of Copenhagen, Copenhagen, Denmark

Thomas Otto *et al.* here presents their long-anticipated analysis of near-full length *de novo* assembled *P. falciparum* *var* genes from genome sequencing of almost 2400 parasite isolates originating from across the word. Understanding the genetic evolution of *var* genes and the organization and variation of the encoded PfEMP1 protein family is an important prerequisite for understanding malaria immunity and pathogenesis. The authors have succeeded in the quite daunting task of condensing the extensive dataset into easily read and interpreted figures explaining their main observations of the gene families organization as well as novel demonstration of var gene sharing and flow linked with spread of drug resistance. The data set itself constitutes an important contribution to the scientific field, and I expect it will form basis of multiple subsequent analyses of car genetic evolution and PfEMP1 function. 

 Through evolution, *var* genes have diversified through acquisition of point mutations and recombination. Despite their extensive diversity, the *var *genes are organized chromosomally, in their recombination patterns and in their encoded domain composition in a manner that is associated and facilitating, functional diversification of the protein family. The higher order of *var* gene division into the UPSB and UPSA genes located on opposite strands in the telomeric regions of the chromosomes and the UPSC genes in the chromosome centromere, and the linkage of this division with the type and phenotype of encoded N-terminal domains (UPSB and UPSC *var* encoded PfEMP1 binds CD36 through their CIDRa domains, UPSA binds EPCR through CIDRa1 or unknown receptors through CIDRg/d/b domains, and the subtelomeric UPSB chimeric B/A type PfEMP1 aka DC8 binds EPCR via CIDRa1), have been described previously, including in several reviews of PfEMP1. Similarly, the typical domain composition of PfEMP1 and PfEMP1 repertoire of each parasite genome, has been described before by the authors. Thus, while these traits are firmly confirmed in the present study, they are understandably not the focus of this study. However, they are key to understand and interpret the observations made throughout the paper, and so I suggest a few simplifications to the introduction as well as some corrections of terminology to ensure consistency with previous papers and help newer readers along (see minor comments below).

 On shared var genes:

 Although the present data assemblies cannot directly infer chromosomal localization of the sequenced genes, most of the genes found to be shared across parasite genomes appear to be centromeric UPSC genes (perhaps calculate and give the proportion of the 11,054 shared genes with high similarity to known centromeric genes of the 15 pacbio reference genomes). As the data suggest, for unknown reasons, these var genes may not recombine as frequently as telomeric var genes, or at least that is as possible explanation for the unexplained conservation of the *var* genetic signature linked to the drug selective sweeps.

 On Recombination:

 The mechanism of var gene recombination has been the center of several studies incl. the referred paper by Sander *et al. *(2014) ^1^, in which chimeric genes were observed in genetic cross progeny clones resulting from ectopic mitotic recombination, i.e. new var genes were results of recombination between var genes of different chromosomes within the same parasite clone (no meiotic rec. events was found in this study). Characteristic of the chimeric genes was multiple closely spaced homologous recombination events. Apart from sequence homology, homologous recombination requires proximity of recombining genes and an initiating event such as DNA strand break or stalling of transcriptional or replication forks (reference) which can cause template switching such as the one observed in the mitotically recombined var genes. The observation that DNA regions with high propensity to form DNA secondary structures (DSS) were condensed around recombination hotspots, in variable sequence regions of var genes and predominantly in recombining PfEMP1 domains, lead to the hypothesis that var genes recombine through homologous recombination initiated by replication-impeding DSS and and facilitated by bouquet formations of chromosome ends during cell replication Freitas-Junior *et al.* (2000) ^2^.

 The identification of recombination sites, in the present study will probably not identify the shortly distanced recombination sites similar to those observed in the parasite cross progeny, and would include meiotic recombination events as well. In addition, the recombination events found appear to be dominated by events within UPSC genes, which appear to recombine less frequently and possibly by a different meitotic mechanism. Altogether, this may explain the suggested discrepancy between this and the Sander *et al*. study in linking DSS to var recombination. However, as homologous recombination sites following replication fork stalling can occur long distances from the initiating event, I would guess the present extraction of recombination sits should capture many of the events leading to the previously determined recombination hotspots and the proposed link to DSS.

 Indeed the authors find that specific recombination sites are not located randomly in the genes, but found in “Hotspots” on the specific gene, which again is non-randomly located, found in particular locations in the var genes, e.g. in the DBLd domains. The data presentation does not allow precise evaluation whether the gene variant specific hotspots aggregate around a specific location in the genes in general e.g. the “mid var genes”, at the DBLd 5´ end. However, the identification of a few short DNA motifs that are common near the majority of recombination sites shows that this is probably the case. A quick search of the localization of the most frequent DNA motifs shows that indeed these are found at the previously identified hotspots of var gene recombination inferred from domain and subdomain homology breaks, i.e. at the 5´end of DBLd domains and the boarder between DBLa subdomain 2 and 3 (MEME motif #2 appears to encode homology block 2 of DBLa, a conserved functional site in DBL domains). Please provide an analysis or statement whether this is true, as this is would be an important result in itself to be highlighted.

 This will also allow the authors to refer to at least these hotspots association with DSS positions, as the two hotspots are the exact same locations which was associated with high frequencies of DSS in Sander *et al*. It is possible that the authors could not find this association as the exact recombination sites +/- 50 bps is skewed somewhat from the hotspots used in Sander *et al.* (HB2 and var mid-point), or that adding meiotic recombination events disturbs the analysis. However, DSS mathematics around these strikingly similar sites should add up. It is unclear what the use of the reference sequences were and in relation to figure S4 showing examples of UPSC var gene hotspots. The authors do not report any statistical test or method, and appear not to have applied the same statistics as Sander *et al.* to test positional association of high propensity DSS with the newly defined recombination hotspots. In Sander *et al.* a cutoff for lowest energy DSS was applied (e.g. the 1 ^st^ or 3 ^rd^ percentile of the 50mers with strongest prediction of forming DSS), and the distance of these DSS was tested for proximity association with the observed recombination hotspots.

 In any case, it is unclear which specific role the authors suggest that the conserved sequence motifs should play in recombination, if not as landmarks to the general hotspots?

 On domains and domain cassettes:

 Each main PfEMP1 domain type exhibits its own distribution of sequence diversity. These domain specific differences in sequence diversity homogeneity and the fact that many domain subclass intermediates exist, makes unbiased and strict domain classification difficult. In the lack of better and to give an operational nomenclature, Rask *et al.* ^3^ applied a simple empirical rule to name all domain possible classes, which was to assign a domain subclass to any group of whole domain sequences containing at least 3 of 7 genomes investigated using maximum likelihood trees. As the present data very nicely show, this approach is not ideal for recombining genes, but still robust for some domain classes, again probably reflecting the different recombination frequencies within these.

 I think the authors have made a wise choice in not attempting to re-name the domain classes, and instead provide beautiful t-SNE plots of each main domain class. This will ease future studies of domain function. For this, I appeal to the authors to include a fasta file with each domain DNA sequence annotated with its “suggestive” subclass type domains for the full dataset. Also, if possible please provide high-resolution images of these t-SNE plots to provide better readability of the legend, and move legend away from spots.

 In some cases, the authors understate the robustness of their data. E.g there is no mentioning of CD36 binding CIDR and their clear division from the EPCR binding CIDRa1 in Figure 8 and S7. Legend in Figure S8 states DBLα subtypes does not cluster, although they do into DBLa1 (USPA) and DBL0 (UPSB/C), as started in main text.

 There are some inconsistency in the mentioned length of MEME motifs – 256 six bp motifs stated in main text and Figure S8, whereas Figure S7 states 15-30 bps?

 Minor comments and suggestions:

1. There is an inconsistent use of “main”, “subclasses”, “subtypes”, “type” and “subdomain classes” in the manuscript.
  - To follow previous terminology I suggest that DBLa,b,g,d,z and CIDRa,b,g,d domians are referred to as “main” classes; e.g. in the sentence “The DBL and CIDR regions have been divided into few main classes DBLa,b,g,d,z and CIDRa,b,g,d based on sequence similarity.” Or alike.
  - I suggest not to use the term “subdomain” but instead use domain “subclass” of the main DBL classes. In PfEMP1, and other proteins, subdomains refer to a smaller functional unit within the domain. E.g. the DBL domain structure is conventionally divided into three subdomains, S1-3., Singh *et al. *(2006) ^4^.
2. The sentence: “Analysis of many thousands of such amplicons first produced a classification system for members of this domain into six classes based on cysteine content, amplicon length and the presence of certain sequence motifs10. Surprisingly, considering the fact that these amplicons only represented a tiny fraction of the total gene sequence, associations have been found with the expression of particular DBLα types and parasite phenotype11,12.”…..is not helpful to the reader. The DBLa tag nomenclature is not used further in the paper, and in fact the ambiguous linkage between Cys2/group A-like DBLa domains and EPCR and non EPCR binding CIDR domains in group A and Cys4 group B like DBLa0 tags and CD36 and EPCR binding in group B and C genes, may appear confusing to readers. I suggest the paragraph is used to present the functional diversification of the majority of PfEMP1 into those binding CD36 via and those binding EPCR via their specific variants of N-terminal CIDR domains, also associated with chromosomal var location, which will help interpretation and understanding of the distribution of genetic diversity presented later.
3. And in the following paragraph: I do understand the need to present the domain cassette idea. However, this could perhaps be introduced later under the section investigating putative domain cassettes or changed not to relate to EPCR/or to reflect current it has been clarified that the EPCR binding phenotype is not tied specifically to the DC8 and DC13 domain cassettes, defined from the 7 first analysed genomes Lau *et al.* (2015) ^5^.
  For molecular evolution studies and for developing phenotype specific molecular tools the domain cassette concept has been valuable, and I think the present study's clarification of the consistency or rather inconsistency of the previously defined domain cassettes is a welcoming opportunity to clearly stipulate that many of these cassettes (outside var1-3, DBLa-CIDR, DBLd-CIDR, perhaps DC5) are more likely results of recent genetic inheritance than functional units.
4. I suggest correcting the sentence: “gene….: *var1CSA* is expressed late in the cell cycle and does not appear to reach the red cell surface22” to “ *var1CSA* is expressed throughout the cell cycle and its product does not appear to reach the red cell surface.” ( *Var1 *does not appear to have a late stage specific expression but rather a cell cycle detached expression).
5. I suggest changing “The latter are evaded through transcriptional switches among the var gene family such that new sequences are periodically expressed. Through their role in immune evasion they have evolved to be extremely polymorphic” to “On the red cell surface, PfEMP1 proteins mediate both adherence to endothelium and are targets of host protective antibodies. To escape immune recognition the PfEMP1 family have evolved to be extremely polymorphic and the parasites can switch var gene expression during asexual replication.” – or similar clarification/simplification.
6. I don’t think the conclusion can be drawn that “functional diversification is not yet complete” from the observation that a domain class is highly recombinogenic. This somehow implies that the parasite is actively seeking for receptors to bind or alike, which does not fit with ancient nature of the protein family. High diversity or recombination rates within specific domain classes, may be their prime function, or it could be that variants have been selected for function and indeed are functional within the sequence space of a long gone settled steady state of recombination (last paragraph page 10).
7. Likewise I suggest rephrasing: “the conclusion that some domain types are now relatively stable while others are still evolving” – to “the conclusion that some domain types are relatively stable while others are highly recombinogenic”.

I confirm that I have read this submission and believe that I have an appropriate level of expertise to confirm that it is of an acceptable scientific standard, however I have significant reservations, as outlined above.

## References

[1] : DNA secondary structures are associated with recombination in major Plasmodium falciparum variable surface antigen gene families. *Nucleic Acids Res*.2014;42(4) : 10.1093/nar/gkt1174 2270-81 10.1093/nar/gkt1174 24253306PMC3936766

[2] : Frequent ectopic recombination of virulence factor genes in telomeric chromosome clusters of P. falciparum. *Nature*.2000;407(6807) : 10.1038/35039531 1018-22 10.1038/35039531 11069183

[3] : Plasmodium falciparum erythrocyte membrane protein 1 diversity in seven genomes--divide and conquer. *PLoS Comput Biol*.2010;6(9) : 10.1371/journal.pcbi.1000933 10.1371/journal.pcbi.1000933 20862303PMC2940729

[4] : Structural basis for Duffy recognition by the malaria parasite Duffy-binding-like domain. *Nature*.2006;439(7077) : 10.1038/nature04443 741-4 10.1038/nature04443 16372020

[5] : Structural conservation despite huge sequence diversity allows EPCR binding by the PfEMP1 family implicated in severe childhood malaria. *Cell Host Microbe*.2015;17(1) : 10.1016/j.chom.2014.11.007 118-29 10.1016/j.chom.2014.11.007 25482433PMC4297295

# Peer review: Reviewer response for version 1

**DOI:** 10.21956/wellcomeopenres.17073.r37259

**Published:** 2019-12-11

**Authors:** Kirk W. Deitsch

**Affiliations:** 1 Department of Microbiology and Immunology, Weill Cornell Medical College, Cornell University, New York, NY, USA

The submission by Otto and colleagues describes a long-awaited study on the evolution of the *var *gene family in the *Laverania *lineage of malaria parasites. The analysis is a tour de force, including var gene sequences from a huge number of parasites isolated from all over the world. The massive number of sequences enabled the authors to infer evolutionary relationships within the gene family and the existence of universally conserved *var *genes. In addition, by examining the flow of *var* gene sequences between parasites, the authors could clearly identify selective sweeps that have made their way through global parasite populations, including recent selective sweeps caused by the spread of artemisinin resistance. The paper describes a valuable resource and a comprehensive analysis of this important gene family that has implications for public health as well as our understanding of parasite evolution. Below are comments regarding a few issues of clarity and presentation.

 Figure 1a provides a nice visual representation of the degree of sequence conservation across chromosome 4 comparing isolate PF0389 with the reference 3D7. The polymorphic nature of the var gene clusters is quite easily observed. However, for the non-aficionado a couple of aspects of the figure are not clear. Specifically, what is shown on the vertical axis (there is no scale)? What are the sharp spikes in sequence conservation that are found flanking the “left” subtelomeric region and the internal var clusters? These regions appear to be much more conserved than even the core genome, although without any scale it is difficult to say how much more conserved. A more precise description might be available in the methods section, although a short description here would be helpful.

 The authors provide interesting data regarding *var1csa*, in particular identifying two distinct alleles and confirming its conservation throughout the globe. This gene has been a bit mysterious since it was first described. The gene is annotated as a pseudogene in 3D7 due to its premature truncation, and it similarly displays frame-shift mutations (often in exon 2) in the full gene sequences of numerous isolates previously published by this group. Do the data from this study enable the authors to determine if the gene typically (or universally) displays frame-shift mutations in its open reading frame? The intron of *var1csa *is similarly short and appears to be missing the region associated with transcription of noncoding RNAs that have been implicated in var gene transcriptional regulation. Is this similarly a universal characteristic of this gene?

 Figure 4 provides a beautiful display of *var *gene sharing as a result of the development and spread of artemisinin resistance in SE Asia. This figure (along with figure 3) demonstrates visually how drug resistance spread. While beyond the scope of the current report, I am curious if there are additional analyses that could be relatively easily derived using this approach. For example, is there additional information associated with each isolate that would enable the authors to incorporate into figure 4 the precise geographic location from which each isolate was obtained? In other words, could these data be used to show where resistance first arose and how it spread throughout SE Asia? Similarly, drug treatment failure in SE Asia is often associated with failure of the partner drug that is supplied with artemisinin, most frequently piperaquine. Would analysis of piperaquine resistance likely show a similar display or would there be a different pattern?

 Figure 7 displays Meme motifs, domain and DBL types as a single figure. The image shows clustering, however this was not initially intuitive to me. This might be easier to understand if a cluster tree was displayed on the left as is often done with heatmaps. More importantly, the dotted box near the top of the figure is described as showing that DBLe4 and DBLe10 show significant similarities and might better be considered as a single subdomain. I gather this conclusion comes from the organizations of the DBL types in blue on the right of the figure. However, the top dotted box seems to include DBLe10 with DBLe3 rather than DBLe4. Is this an error in how the labels on the horizontal axis were aligned?

I confirm that I have read this submission and believe that I have an appropriate level of expertise to confirm that it is of an acceptable scientific standard.
